# Bubble NIPPV: Guidelines for Use

**DOI:** 10.3390/children12070834

**Published:** 2025-06-25

**Authors:** Mounika Muttineni, Vineet Bhandari, Stephen John, Tina Slusher

**Affiliations:** 1 Department of Pediatrics, Children’s National Hospital, Washington, DC 20010, USA; mmuttineni@childrensnational.org; 2 Department of Pediatrics, Cooper Medical School of Rowan University, Camden, NJ 08103, USA; 3 Department of Pediatrics, The Children’s Regional Hospital at Cooper, Camden, NJ 08103, USA; 4 Department of Internal Medicine, University of Texas Health Science Center at Houston, Houston, TX 77030, USA; stephen.c.john@uth.tmc.edu; 5 Department of Pediatrics, University of Minnesota Medical Center, Minneapolis, MN 55455, USA; tslusher@umn.edu

**Keywords:** newborn, preterm infant, NCPAP, bubble CPAP, NIPPV, bubble NIPPV, non-invasive ventilation

## Abstract

Neonatal respiratory distress is a primary contributor to neonatal morbidity and mortality worldwide. Non-invasive respiratory support such as nasal continuous positive airway pressure (NCPAP) and bubble NCPAP (bNCPAP) are often used as the first line of treatment for neonatal respiratory distress, including respiratory distress syndrome; however, many hospitals in low- and middle-income countries do not have access to advanced respiratory support devices beyond NCPAP. A novel, non-invasive bubble positive pressure ventilation device has been developed as a low-cost, non-electric alternative to providing respiratory support in such scenarios. In this article, we propose evidence-based guidelines for the initiation, titration, and weaning of the new device.

## 1. Introduction

Almost 2.5 million infants die every year within the first month of life, with low- and middle-income countries (LMICs) bearing the heaviest burden. The leading causes are prematurity, birth complications, and infections—all of which can lead to respiratory distress and subsequent respiratory failure [1]. Despite a decrease in overall child mortality, current global trends reveal an increase in the proportion of neonatal deaths among all under-five deaths, from 40% in 1990 to 47% in 2021 with South Asia and Sub-Saharan Africa carrying the highest numbers [2]. Over 90% of infants with extreme prematurity (<28 weeks) born in LMICs die within the first few days of life, as opposed to <10% in high-income countries (HICs) [3]. Therefore, improving the management of preterm respiratory distress in LMICs is imperative to reducing neonatal death rates.

Non-invasive respiratory supports, such as nasal continuous positive airway pressure (NCPAP) and bubble nasal CPAP (bNCPAP), have long been accepted to be an effective form of first-line therapy for respiratory distress in neonates, including secondary to respiratory distress syndrome (RDS) in preterm infants. In high-resource settings, invasive mechanical ventilation (IMV) has been associated with the development of lung inflammation, leading to bronchopulmonary dysplasia (BPD) and associated neurological sequelae [4]. In low-resource settings, the additional burden of high patient-to-nurse ratios and limited access to blood gas equipment make invasive ventilation even less desirable. Thus, there is strong motivation to limit the need for invasive ventilation in these settings. Growing evidence supports the use of nasal intermittent positive pressure ventilation (NIPPV) as being superior to NCPAP in primary and post-extubation support for sicker infants, with lower rates of respiratory failure necessitating intubation or post-extubation failure in comparison with NCPAP [5,6]. Some data also show decreased BPD rates with the use of NIPPV [7]. The benefits of NIPPV can be attributed to improved oxygenation due to a higher mean airway pressure and improved ventilation due to the pressure gradient created by the difference in higher and lower pressures [8,9,10,11].

Ventilator-based NIPPV protocols have been established, outlining criteria for initiation, initial parameter selection, adjustment of parameters, and weaning of therapy [12]. It is evident that NIPPV is the preferred mode of primary and secondary respiratory support in infants with RDS, but availability, expense, and lack of appropriate training limit its feasibility in LMICs [13]. Moreover, significant barriers of cost, equipment complexity, access to replacement parts/servicing, and lack of continuous electrical power prevent many resource-constrained settings from adopting ventilator-based NIPPV.

A novel, simple bubble NIPPV (bNIPPV) machine has been developed, which utilizes the same operating principle as bNCPAP, wherein the submerged depth of the circuit’s expiratory limb sets the delivered pressure through a hydrostatic principle [14]. As gas passes through water, it creates bubbles, generating pressure oscillations that facilitate gas exchange and lung alveoli recruitment [15]. This technology is simple, non-electric, and economical. It has thus far been demonstrated to be feasible [16] to use in an under-resourced setting with a similar safety profile to bNCPAP [17]. The efficacy of this mode of non-invasive support has yet to be studied in sufficiently powered randomized clinical trials (RCTs). Given the need to do so, it would be important to provide information and guidelines for the use of bNIPPV. In this article, we review the existing literature for the use of ventilator-based NIPPV and provide guidelines for the use of bNIPPV initiation, adjustment, and weaning as a mode of initial and post-extubation therapy.

## 2. Nomenclature

NCPAP provides a constant positive end expiratory pressure (PEEP). Pressure is generated by a ventilator, a flow driver, or an underwater seal. When pressure is generated by an underwater seal, or a bubble system, it is referred to as bNCPAP. NIPPV is a system that combines PEEP with intermittent peak inspiratory pressures (PIP), and similarly, bNIPPV utilizes an underwater seal for its pressure generation [18]. There are two major settings in which NIPPV can be used, which are referred to as primary and secondary modes. The primary mode of NIPPV describes its use soon after birth with or without a short period of intubation for the sole purpose of surfactant delivery and subsequent extubation. The secondary mode refers to its use following a longer period of intubation [12]. NIPPV is often confused with BiPAP or bi-level NCPAP, both of which have two pressure settings. The main differences include lower peak pressures with BiPAP and bi-level NCPAP (e.g., P high or PIP of 8 cm H_2_O, P low or PEEP of 5 cm H_2_O). Additionally, for the bi-level NCPAP, there is a longer duration at the higher pressure (inspiratory time or Ti) as compared to NIPPV [19,20].

### 2.1. Bubble NIPPV Equipment Description and Use

**Figure 1:**
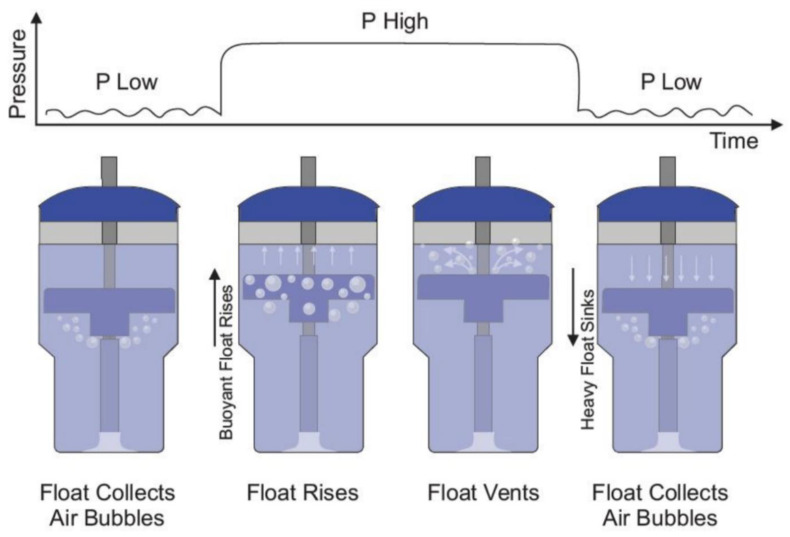
Bubble NIPPV’s mechanism of action. Figure used with permission from AIM Tech.

bNCPAP and bNIPPV utilize the same infant respiratory circuit in which a continuous baseline pressure is set by the submerged depth of the circuit’s expiratory limb. In contrast to bNCPAP where a constant level of expiratory pressure is delivered, bNIPPV delivers two different levels of pressure: PIP (8 to 20 cm H_2_O) and PEEP (5 to 8 cm H_2_O). To accomplish this, the bNIPPV utilizes a novel and non-electric variable buoyancy float to cycle the level of pressure. Bubbles from the expiratory gas flow are collected in the variable buoyancy float. As the float accumulates these bubbles, it becomes buoyant and rises to the top of the container. This motion blocks further air escape from the bubbling outlet, instead forcing the air to leave the closed circuit through pressure-regulating valves generating PIP. As the float vents the collected bubbles, it loses its buoyancy and sinks, reopening the bubbling outlet producing PEEP and returning the system to its original state (Figure 1). The submerged vertical tube contains distal venting holes, which serve as a safety measure, limiting the maximum delivered pressure in the event of a pressure valve failure.

The frequency at which the float oscillates is directly proportional to the rate of flow of compressed air/oxygen. In other words, as the flow rate is increased from 5 to 10 LPM, the float fills with air more rapidly and rises sooner, leading to a shorter duration at the low-pressure level and thereby shortening the overall cycle time [21].

bNIPPV is not synchronized with the patient’s efforts. However, anecdotally, patients seem to synchronize their respirations with the delivered pressure cycles, as has also been noted with infants on ventilator-driven non-synchronized NIPPV (nsNIPPV).

**Figure 2:**
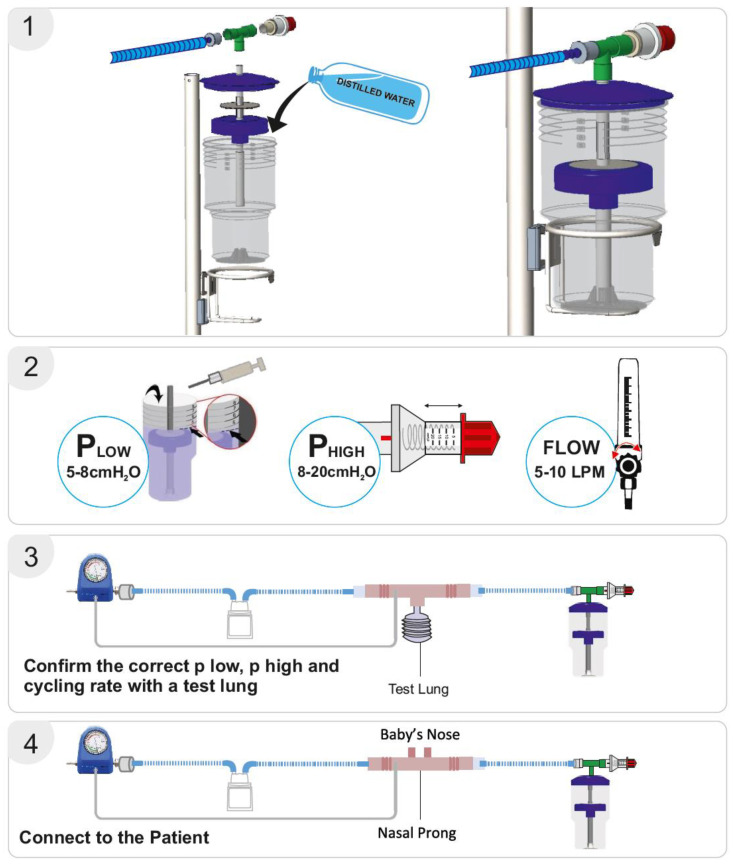
Bubble NIPPV set up. Figure used with permission from Phoenix Medical Systems. Subfigure (**1**) illustrates the proper assembly of parts. Subfigure (**2**) shows how to set the P_low_ or PEEP, and P_high_ or PIP. Subfigures (**3**,**4**) depict the whole circuit connected to a test lung or an infant nose respectively, with verification of delivered pressures achieved through the attached pressure monitor. Video link for guidance: https://youtu.be/zoNXS5GUOzs, accessed on 12 May 2025.

The bNIPPV unit consists of a container, central pipe, float, T-connector, and pressure regulator. The container is filled with distilled water to set the desired low-pressure level as indicated by respective markings on the container. Thereafter, the float is placed on the central pipe, and this pipe is then inserted into the container. The lid is placed on the container, after which the T-connecter is attached to the pressure regulator, central pipe, and respiratory circuit. The high pressure can be set by adjusting this pressure regulator. As in the case of bNCPAP, a blended mixture of compressed air and oxygen is passed through a heated humidifier (if available) to the inspiratory limb of the breathing circuit. The flow of air/oxygen mixture must be set to at least 5–10 L/min (Figure 2). As discussed previously, the expiratory limb of the breathing circuit is connected to the bNIPPV device.

**Figure 3:**
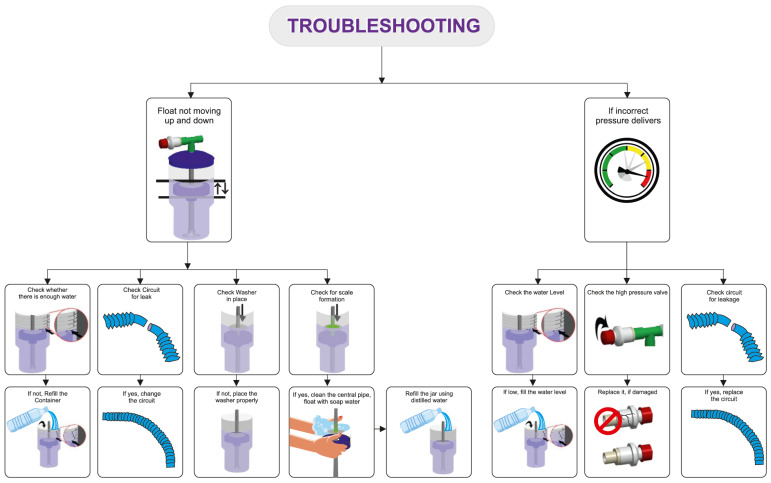
Troubleshooting common bubble NIPPV issues. Figure used with permission from Phoenix Medical Systems.

The delivery of the prescribed low and high pressures is first confirmed with a test lung and an in-line pressure transducer prior to connecting the device to the patient [17]. An occlusive nasal interface should be used to ensure adequate delivery of pressure. Hospitals have used the Hudson prong nasal interface, larger-sized RAM cannula, Fisher & Paykel, and Dräger interfaces. If the float does not cycle up and down, there may be a leak in the circuit, inadequate flow of compressed air, or a layer of biofilm between the central pipe/float. If the float is cycling up and down but the prescribed pressures are not delivered, an assessment for an incorrect water fill level, an incorrect setting on the pressure valve, or a leak in the respiratory circuit should be conducted. After the delivery of the prescribed pressures is confirmed with the test lung, the bNIPPV can be connected to the patient. If there is a decrease in delivered pressures to the infant, this likely represents a leak at the nasal interface which should be addressed [22] (Figure 3).

### 2.2. Proposed Guidelines and Settings

**Figure 4:**
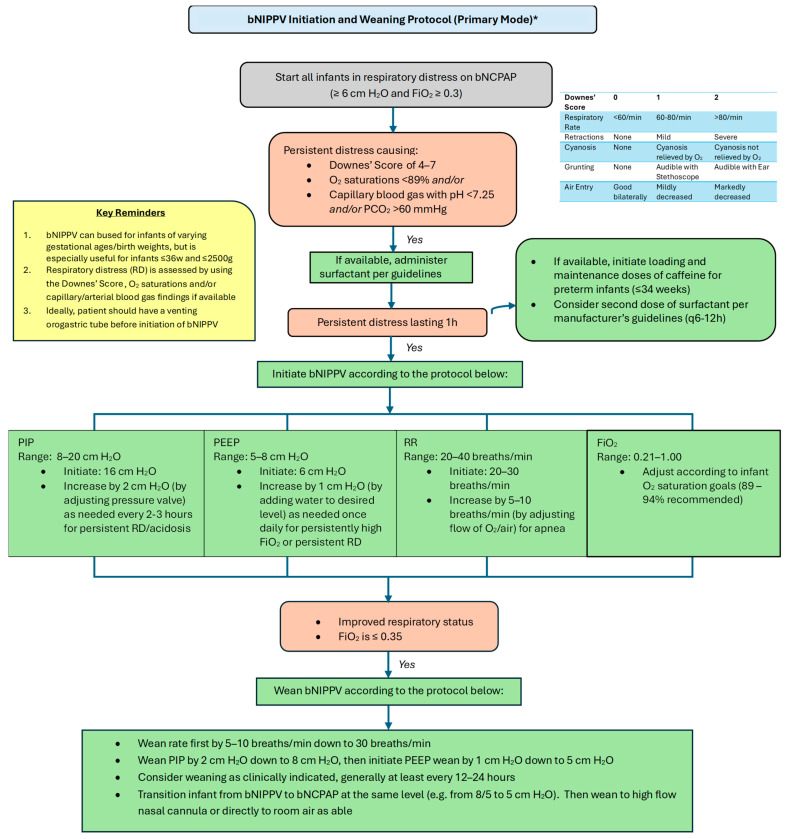
Bubble NIPPV initiation and weaning protocol (primary mode) *. * Primary mode refers to use soon after birth and/or following a short period of intubation for surfactant delivery. bNIPPV: bubble nasal intermittent positive pressure ventilation; bNCPAP: bubble nasal continuous positive airway pressure; PIP: peak inspiratory pressure; PEEP: positive end expiratory pressure; RR: respiratory ventilation rate; FiO_2_: fraction of inspired oxygen.

**Figure 5:**
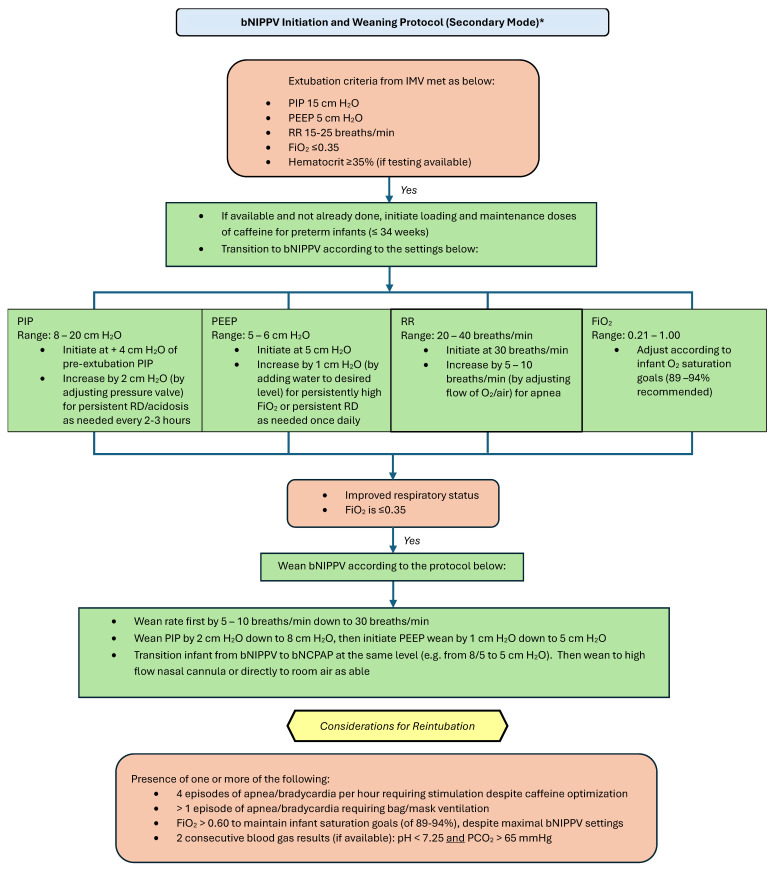
Bubble NIPPV initiation and weaning protocol (secondary mode) *. * Secondary mode refers to use after a longer period of intubation. IMV: invasive mechanical ventilation; bNIPPV: bubble nasal intermittent positive pressure ventilation; bNCPAP: bubble nasal continuous positive airway pressure; PIP: peak inspiratory pressure; PEEP: positive end expiratory pressure; RR: respiratory (ventilator) rate; FiO_2_: fraction of inspired oxygen.

Please refer to Figure 4 and Figure 5 for our proposed guidelines and settings for the usage of bubble NIPPV in the primary and secondary modes, including surfactant administration [23,24].

## 3. Discussion

Respiratory failure is the most common cause of neonatal death worldwide. The European Consensus Guidelines on the Management of Respiratory Distress Syndrome recommend non-invasive ventilation as the ideal support for preterm infants with RDS [25]. (2022 Update). NCPAP is often the first form of support attempted in this setting with failure necessitating invasive ventilation at a rate of 45% to 60% [26]. NIPPV has emerged as a superior mode of respiratory support in preventing the need for IMV, decreasing the incidence of post-extubation failure, and potentially minimizing the risk of BPD [5,27,28].

In a systematic review and network meta-analysis performed by Ramaswamy and colleagues (2020), non-synchronized BiPAP (BiPAP), nsNIPPV, and synchronized (sNIPPV) all decreased the risk for IMV and BPD occurrence compared to NCPAP [28]. In this article, we review RCTs that compare the efficacy of NIPPV, BiPAP, or both modalities with NCPAP in neonates with RDS as utilized in primary and secondary modes. These trials were chosen based on inclusion in two large systematic reviews examining the use of NIPPV in neonatal RDS, one published by Clinics in Perinatology [5] and the other by the Cochrane Collaboration [6,28]. Notably, these trials include significantly different study populations and employ varied settings and equipment to deliver respiratory support as highlighted in Table 1, Table 2 and Table 3.

Table 1 summarizes RCTs that study the efficacy of ventilator-driven NIPPVs in comparison with NCPAP in primary and secondary modes. In general, more favorable outcomes were observed in the NIPPV arms without any notable adverse events. Table 2 details RCTs that examine BiPAP vs. NCPAP efficacy in primary and secondary modes. These studies overall found less significant differences between the two modalities, which may be attributable to the lower pressures used in BiPAP mode when compared to NIPPV. Table 3 contains RCTs, which incorporate both NIPPV and BiPAP in the experimental group as opposed to a singular mode. The largest multi-centered RCT led by Kirpalani et al. on the comparison of NCPAP and NIPPV in the primary and secondary modes concluded that there was no difference in the rate of intubation, re-intubation, and survival to 36 weeks postmenstrual age without BPD in the very-low-birth-weight infants. This is perhaps reflective of the varied settings and modalities used in the experimental arm, as ~53% of this arm employed BiPAP settings.

Of all the trials analyzed, none found a notable advantage for NCPAP. None of these trials observed a difference in the safety profile of NIPPV and NCPAP. There were initial concerns of gastrointestinal (GI) perforation, although two systematic reviews of the Cochrane Collaboration on NIPPV in the primary and secondary mode have suggested similar rates of GI complications with both NIPPV and NCPAP usage [6]. It is a recommended standard practice to place an abdominal venting device (an 8 or 9 Fr oro-gastric tube, with the proximal part of a 10cc syringe attached to it (plunger removed), kept open to air and at a higher level than the baby) to minimize abdominal distension.

In settings where effective NCPAP has failed and further ventilator-based resources are limited or unavailable, the novel bNIPPV device offers a reliable alternative. Its existing literature has been promising and has been proven to be clinically safe and feasible to use. In infant manikins, the ability to consistently cycle between a set lower pressure and higher pressure with bNIPPV was established [21]. In the IngMar ASL 5000 infant lung simulator, the delivered pressure and volumes of bNIPPV were compared with those of the conventional ventilator-driven NIPPV with lung resistance and compliance set to approximate values of infants with RDS, transient tachypnea of the newborn, and pneumonia [14]. In an animal model, the ability of bNIPPV to normalize arterial blood gas values in sedated rabbits was compared with that of a conventional ventilator [53].

The feasibility of bNIPPV in comparison to bNCPAP was established in a cross-over case series, in which patients were randomized to four hours of bNCPAP vs. bNIPPV, followed by the alternate treatment [16]. This was followed by a formal study of safety, in which 60 infants in respiratory distress were pragmatically allocated to bNCPAP vs. bNIPPV (30 infants in each arm). A similar rate of complications, including septal necrosis, pneumothorax, and gastric distention, was observed in both arms [17]. Notably, this safety study utilized a lower level of PIP (similar to what is used in BiPAP); however, the proposed guidelines are grounded on existing literature surrounding ventilator generated NIPPV usage in the primary and secondary modes as referenced in Table 1, Table 2 and Table 3. While the PIPs recommended for bNIPPV use in this protocol have not been studied in an RCT, the PIPs recommended are still lower based on our experience of >25 years of using ventilator-generated NIPPV and those reported in other studies [10,27,54]. It is important to use such pressures, if needed, in preterm infants with respiratory distress in resource-limited countries (with little or no access to exogenous surfactant), as avoiding intubation and IMV is an important goal because of its potential life-threatening complications, such as sepsis.

In summary, most studies on NIPPV have confirmed its superiority over NCPAP in terms of preventing extubation failure. This aspect of NIPPV is of critical importance in low-resource settings where access to IMV is limited, in addition to the other known complications of IMV (for, e.g., ventilator associated pneumonia and sepsis). Hence, in this scenario where the NCPAP is not effective, the NIPPV has the potential to provide enhanced non-invasive support. Higher initial NIPPV settings may be required to ascertain a greater effect on the prevention of initial IMV as well as the prevention of reintubation following extubation. The novel bNIPPV device—with its associated benefits of being low-cost, nonelectric, and simple to use—offers those infants without access to advanced respiratory support something that bNCPAP alone often fails to do—a chance at survival. These guidelines for the use of bNIPPV provide a thorough and important resource for medical personnel taking care of neonates in LMICs.

## Figures and Tables

**Table 1:** Randomized controlled trials observing the efficacy of ventilator NIPPV vs. CPAP.

					*NIPPV*			*CPAP*			
	Study	n	Type	Mean BW (g) & GA (w) *	PIP (cm H_2_O) ^¥^	PEEP (cm H_2_O) ^¥^	RR (br/min) ^¥^	Type	Mean BW (g) & GA (w)	PEEP (cm H_2_O) ^¥^	Noteworthy NIPPV Outcomes
***Primary Mode***	Bisceglia et al (2007) [29]	88	ventilator, non-synchronized	1020 g, 29.8 w	14–20	4–6	40	n/a	1010 g, 30.6 w	4–6	- no difference in IMV ^1^ occurrence - decreased PCO_2_ - decreased apneic episodes - decreased duration of NIV ^2^ support
	Kishore et al (2009) [30]	76	ventilator, non-synchronized	1250 g, 30.7 w	15–24/26	5–6	50–60	ventilator	1250 g, 30.8 w	5–7	- decreased IMV occurrence in the first 48 HOL ^3^ & 7 d
	Meneses et al. , (2011) [31]	200	ventilator, non-synchronized	1112 g, 29 w	15–20	4–6	20–30	bubble	1151 g, 30.1 w	5–6	- no difference in overall IMV occurrence in first 72 h, but decrease observed in 24–72 HOL
	Ramanathan et al (2012) [32]	110	ventilator, non-synchronized	1052 g, 27.8 w	10–15	5	30–40	mixed	1099 g, 27.8 w	5–8	- decreased IMV occurrence in the first 7 d - decreased BPD ^4^ occurrence
	Armanian et al (2014) [33]	98	ventilator, non-synchronized	1261.4 g, 30.4 w	16–20	5–6	50–60	bubble	1156.5 g, 29.5 w	5–6	- no difference in IMV occurrence - decreased duration of NIV support - decreased duration of O_2_ dependency - faster time to reach full enteral feeds
	Oncel et al (2015) [34]	200	ventilator, non-synchronized	1180 g, 29.2 w	15–20	5–6	20–30	ventilator	1175 g, 29.1 w	5–6	- decreased IMV occurrence in first 72 HOL - decreased surfactant need in first 72 HOL
	Sabzehei et al (2018) [35]	60	ventilator, non-synchronized	1259 g, 30.1 w	14–20	5–6	30–50	ventilator	1235 g, 30.1 w	5–6	- no difference in IMV occurrence - decreased duration of NIV support
	Skariah & Lewis et al (2019) [36]	78	ventilator, non-synchronized	1400 g, 31.8 w	11–18	3–5	18–30	ventilator	1440 g, 31.7 w	3–5	- no difference in IMV occurrence - decreased surfactant need - decreased respiratory distress until 12 HOL - decreased time to full feed
	Shi et al (2021) [37]	179	ventilator, non-synchronized	2421 g, 34.3 w	15–20	4–6	10–20	bubble	2349 g, 34.2 w	4–6	- decreased IMV occurrence - more favorable discharge outcomes
***Secondary Mode***	Khorana et al (2008) [38]	48	ventilator, non-synchronized	948 g, 28.3 w	pre-ext. ^5$^	pre-ext.	pre-ext.	n/a	1067 g, 29.3 w	pre-ext	- no difference in extubation failure rate
	Kahramaner et al (2014) [39]	67	ventilator, non-synchronized	1228 g, 29.3 w	+2 pre-ext. ^ž^	6	25	variable flow	1091 g, 28.1 w	6	- decreased extubation failure rate - decreased post-extubation atelectasis
	Jasani et al (2015) [40]	63	ventilator, non-synchronized	1187 g, 30.8 w	+4 pre-ext. ^‡^	</=5	same as pre-ext.	ventilator /variable flow	1153 g, 30.6 w	5–6	- no difference in extubation failure rate - decreased duration of NIV support - decreased duration of O_2_ dependency - decreased BPD occurrence
	Komatsu et al (2016) [41]	72	ventilator, non-synchronized	1271 g, 30.2 w	16	6	12	ventilator	1425 g, 31.2 w	6	- no difference in extubation failure rate
	Ribeiro et al (2017) [42]	101	ventilator, non-synchronized	1121 g, 29.3 w	14–16 ^^^	4–6	12–18	bubble/ ventilator	1157 g, 29.7 w	4–5	- no difference in extubation failure rate
	Etsay et al (2020) [43]	220	ventilator, non-synchronized	1034 g, 27.8 w	12–15 ^◊^ (<1 kg); 14–18 (>1 kg)	5–6	20	bubble/ ventilator	1019 g, 27.9 w	5–6	- no difference in extubation failure rate

Abbreviations: ^1^ IMV: invasive mechanical ventilation, ^2^ NIV: non-invasive ventilation, ^3^ HOL: hours of life, ^4^ BPD: bronchopulmonary dysplasia, ^5^ pre-ext: pre-extubation. Legend: * numbers rounded to the nearest tenth; ¥ PIP, PEEP, and RR settings reported as initiation to max settings; $ extubation criteria: PIP < 15 cm H_2_O, PEEP < 5 cm H_2_O, rate < 15 breaths/min, FiO_2_ < 0.4; ž extubation criteria: PIP ≤ 14 cm H_2_O, rate 10 breaths/min; ‡ extubation criteria: caffeine load, persistent good resp effort higher than vent rate, normal blood gas on min ventilation (mean airway pressure ≤ 7 cm, FiO_2_ ≤ 35%); ^ extubation criteria: PIP < 16 cm H_2_O, PEEP < 6 cm H_2_O, rate < 18 breaths/min, FiO_2_ < 0.4 with normal blood gases; ◊ extubation criteria: FiO_2_ ≤ 0.5, PIP ≤ 18 cm H_2_O, RR ≤ 20, to maintain a pH ≥ 7.25, oxygen saturation ≥ 88% and a PaCO_2_ ≤ 65 mmHg.

**Table 2:** Randomized controlled trials observing the efficacy of BiPAP vs. CPAP.

	Study	n	Type	Mean BW (g) & GA (w)	PIP (cm H_2_O) ^¥^	PEEP (cm H_2_O) ^¥^	RR (br/min) ^¥^	*CPAP* Type	Mean BW (g) & GA (w)	PEEP (cm H_2_O) ^¥^	Noteworthy BiPAP Outcomes
***Primary Mode***	Kong et al (2012) [44]	67	flow-driver, non-synchronized	1983 g, 32.9 w	12−15	4−6	20−30	constant flow	2036 g, 32.8 w	4−6	- decreased IMV ^1^ occurrence
	Aguiar et al (2015) [45]	220	flow-driver, non-synchronized	1355 g, 31.1 w	8	6	10−15	variable flow	1373 g, 30.9 w	6−8	- no overall difference in IMV occurrence in the first 120 h, but decreased in 30–32.6 w subgroup
	Sadeghnia et al (2016) [46]	70	flow-driver, non-synchronized	1129.1 g, 29.4 w	8	4	30	bubble	1090.57 g, 28.32 w	6	- no difference in IMV occurrence - no difference in duration of O_2_ dependency - no difference in duration of NIV ^2^ support
	Pan et al (2021) [47]	284	flow-driver, non-synchronized	1251 g, 30.1 w	9	5	30	variable flow	1264 g, 29.6 w		- no difference in IMV occurrence - decreased duration of NIV support
***Secondary Mode***	O’Brien et al (2012) [48]	136	flow-driver, non-synchronized	901 g, 27.3 w	8−10 ^§^	5−7	20	variable flow	896 g, 27.4 w	5−7	- no difference in extubation failure rate - increased ROP ^3^
	Victor et al (2016) [49]	540	flow-driver, non-synchronized	870 g, 26 w 1185 g, 29 w	6−8 ^≠^	4	30	variable flow	910 g, 26 w 1173 g, 28 w	4−6	- no difference in extubation failure rate in sequential BiPAP + CPAP vs. CPAP alone
	Manjunatha et al (2019) [50]	119	flow-driver, non-synchronized	~1038 g *, 28−30 w	8−11 ^≈^	5−8	30−40	variable flow	~959 g *, 28−30 w	5−8	- no difference in extubation failure rate 72 h post extubation

Abbreviations: ^1^ IMV: invasive mechanical ventilation, ^2^ NIV: non-invasive ventilation, ^3^ ROP: retinopathy of prematurity retinopathy of prematurity. Legend: ¥ PIP, PEEP, and RR settings reported as initiation to max settings; § extubation criteria: conventional MV: PIP ≤ 16 cm H_2_O and FiO_2_ ≤ 0.35; high frequency MV: frequency 9–13 Hz, amplitude < 20 percent mean airway pressure ≤ 8 cm H_2_0, FiO_2_ ≤ 0.35; ≠ extubation criteria: (PIP) ≤ 15 cm H_2_O, positive end expiratory pressure (PEEP) 5 cm H_2_O, rate of 15–25, FiO_2_ of 0.35 and hematocrit of > 35% * rough estimates based on information given; ≈ extubation criteria: minimal ventilation requirements, good respiratory effort with hematocrit >35%.

**Table 3:** Randomized controlled trials observing the efficacy of both NIPPV/BiPAP vs. CPAP.

		Study	n	Type	Mean BW (g) & GA (w)	*NIPPV/BiPAP* PIP (cm H_2_O) ^¥^	PEEP (cm H_2_O) ^¥^	RR (br/min) ^¥^	*CPAP* Type	Mean BW (g) & GA (w)	PEEP (cm H_2_O) ^¥^	Noteworthy BiPAP/NIPPV Outcomes
***Secondary Mode***	***Primary Mode***	Kirpalani et al (2013) [51]	1009	~53% flow-driver BiPAP, remainder ventilator NIPPV	802 g, 26.1 w	15–18 or +2–4 pre-ext. ^◊^	5–8 or same as pre-ext.	10–40	mixed methods	805 g, 26.2 w	5–6 or same as pre-ext.	- no difference in the incidence of intubation - no difference in extubation failure rate - no difference in BPD ^1^ occurrence
		El Farrash et al (2022) [52]	120	ventilator NIPPV, ventilator BiPAP	1940 g, 32.7 w (NIPPV) 1700 g, 32.1 w (BiPAP)	MAP 7 cm H_2_O ^÷^			ventilator	1810 g, 32.9 w	MAP 7 cm H_2_O	- no difference in extubation failure rate - increased duration of hospitalization & duration of IMV ^2^ in BiPAP group only

Abbreviations: ^1^ BPD: Bronchopulmonary Dysplasia, ^2^ IMV: invasive mechanical ventilation. Legend: ¥ PIP, PEEP, and RR settings reported as initiation to max settings; ÷ Extubation criteria: (1) blood gas with pH > 7.25 and capillary pCO_2_ < 52.5 mm Hg, (2) MAP 7 cm H_2_O, (3) FiO_2_ < 0.35, (4) persistent, spontaneous RR > present ventilator rate, (5) caffeine load; ◊ extubation criteria: none specifically reported.

## Data Availability

Not applicable.

## References

[1] Kamath B.D., Macguire E.R., McClure E.M., Goldenberg R.L., Jobe A.H. (2011). Neonatal mortality from respiratory distress syndrome: Lessons for low-resource countries. Pediatrics.

[2] United Nations Children’s Fund Neonatal Mortality. https://data.unicef.org/topic/child-survival/neonatal-mortality/.

[3] Ekhaguere O.A., Okonkwo I.R., Batra M., Hedstrom A.B. (2022). Respiratory distress syndrome management in resource limited settings-Current evidence and opportunities in 2022. Front. Pediatr..

[4] Bhandari A., Carroll C., Bhandari V. (2016). BPD Following Preterm Birth: A Model for Chronic Lung Disease and a Substrate for ARDS in Childhood. Front. Pediatr..

[5] Ruegger C.M., Owen L.S., Davis P.G. (2021). Nasal Intermittent Positive Pressure Ventilation for Neonatal Respiratory Distress Syndrome. Clin. Perinatol..

[6] Lemyre B., Deguise M.O., Benson P., Kirpalani H., Ekhaguere O.A., Davis P.G. (2023). Early nasal intermittent positive pressure ventilation (NIPPV) versus early nasal continuous positive airway pressure (NCPAP) for preterm infants. Cochrane Database Syst. Rev..

[7] Bhandari V., Gavino R.G., Nedrelow J.H., Pallela P., Salvador A., Ehrenkranz R.A., Brodsky N.L. (2007). A randomized controlled trial of synchronized nasal intermittent positive pressure ventilation in RDS. J. Perinatol..

[8] Mukerji A., Abdul Wahab M.G., Razak A., Rempel E., Patel W., Mondal T., Beck J. (2021). High CPAP vs. NIPPV in preterm neonates—A physiological cross-over study. J. Perinatol..

[9] Owen L.S., Morley C.J., Davis P.G. (2007). Neonatal nasal intermittent positive pressure ventilation: What do we know in 2007?. Arch. Dis. Child. Fetal Neonatal Ed..

[10] Shi Y., Muniraman H., Biniwale M., Ramanathan R. (2020). A Review on Non-invasive Respiratory Support for Management of Respiratory Distress in Extremely Preterm Infants. Front. Pediatr..

[11] Boel L., Broad K., Chakraborty M. (2018). Non-invasive respiratory support in newborn infants. Paediatr. Child Health.

[12] Bhandari V. (2010). Nasal intermittent positive pressure ventilation in the newborn: Review of literature and evidence-based guidelines. J. Perinatol..

[13] Kumar J., Kumar P., Bhandari V. (2025). Noninvasive ventilation strategies in neonates. Indian Pediatr..

[14] John S.C., John A.V., Moss A.W., Gustafson P.A., Fernando-Silva L., John S.P. (2020). Bench Testing of a Bubble Noninvasive Ventilation Device in an Infant Lung Simulator. Respir. Care.

[15] Poletto S., Trevisanuto D., Ramaswamy V.V., Seni A.H.A., Ouedraogo P., Dellaca R.L., Zannin E. (2023). Bubble CPAP respiratory support devices for infants in low-resource settings. Pediatr. Pulmonol..

[16] John S.C., Adhikari B.R., John A.V., Cheng E.O., Weiner G.M., John S.P. (2022). Feasibility of bubble non-invasive positive pressure ventilation, a first-in-human study. J. Trop. Pediatr..

[17] John S.C., Garg M., Muttineni M., Brearley A.M., Rao P., Bhandari V., Slusher T., Murki S. (2024). Safety of bubble nasal intermittent positive pressure ventilation (NIPPV) versus bubble nasal continuous positive airway pressure (NCPAP) in preterm infants with respiratory distress. J. Perinatol..

[18] Falk M., Donaldsson S., Drevhammar T. (2018). Infant CPAP for low-income countries: An experimental comparison of standard bubble CPAP and the Pumani system. PLoS ONE.

[19] Bhandari V. (2017). NIPPV in the neonate: Answers to FAQs—A personal perspective. J. Neonatol..

[20] Owen L.S., Manley B.J. (2016). Nasal intermittent positive pressure ventilation in preterm infants: Equipment, evidence, and synchronization. Semin. Fetal Neonatal Med..

[21] John S.C., Barnett J.D., Habben N.D., Le H.T., Cheng E., John S.P., Gustafson P.A. (2017). Development and Testing of a Bubble Bi-Level Positive Airway Pressure System. Respir. Care.

[22] John S.C., Cheng E.O., John S.P. (2020). The BCPAP Score: Five Questions to Assess the Effectiveness of a Bubble CPAP Circuit. J. Trop. Pediatr..

[23] Downes J.J., Vidyasagar D., Boggs T.R., Morrow G.M. (1970). Respiratory distress syndrome of newborn infants. I. New clinical scoring system (RDS score) with acid–base and blood-gas correlations. Clin. Pediatr..

[24] Bhandari V., Black R., Gandhi B., Hogue S., Kakkilaya V., Mikhael M., Moya F., Pezzano C., Read P., Roberts K.D. (2023). RDS-NExT workshop: Consensus statements for the use of surfactant in preterm neonates with RDS. J. Perinatol..

[25] Sweet D.G., Carnielli V.P., Greisen G., Hallman M., Klebermass-Schrehof K., Ozek E., Te Pas A., Plavka R., Roehr C.C., Saugstad O.D. (2023). European Consensus Guidelines on the Management of Respiratory Distress Syndrome: 2022 Update. Neonatology.

[26] Wright C.J., Sherlock L.G., Sahni R., Polin R.A. (2018). Preventing Continuous Positive Airway Pressure Failure: Evidence-Based and Physiologically Sound Practices from Delivery Room to the Neonatal Intensive Care Unit. Clin. Perinatol..

[27] Ramaswamy V.V., More K., Roehr C.C., Bandiya P., Nangia S. (2020). Efficacy of noninvasive respiratory support modes for primary respiratory support in preterm neonates with respiratory distress syndrome: Systematic review and network meta-analysis. Pediatr. Pulmonol..

[28] Lemyre B., Deguise M.O., Benson P., Kirpalani H., De Paoli A.G., Davis P.G. (2023). Nasal intermittent positive pressure ventilation (NIPPV) versus nasal continuous positive airway pressure (NCPAP) for preterm neonates after extubation. Cochrane Database Syst. Rev..

[29] Bisceglia M., Belcastro A., Poerio V., Raimondi F., Mesuraca L., Crugliano C., Corapi U.P. (2007). A comparison of nasal intermittent versus continuous positive pressure delivery for the treatment of moderate respiratory syndrome in preterm infants. Minerva Pediatr..

[30] Kishore M.S., Dutta S., Kumar P. (2009). Early nasal intermittent positive pressure ventilation versus continuous positive airway pressure for respiratory distress syndrome. Acta Paediatr..

[31] Meneses J., Bhandari V., Alves J.G., Herrmann D. (2011). Noninvasive ventilation for respiratory distress syndrome: A randomized controlled trial. Pediatrics.

[32] Ramanathan R., Sekar K.C., Rasmussen M., Bhatia J., Soll R.F. (2012). Nasal intermittent positive pressure ventilation after surfactant treatment for respiratory distress syndrome in preterm infants <30 weeks’ gestation: A randomized, controlled trial. J. Perinatol..

[33] Armanian A.M., Badiee Z., Heidari G., Feizi A., Salehimehr N. (2014). Initial Treatment of Respiratory Distress Syndrome with Nasal Intermittent Mandatory Ventilation versus Nasal Continuous Positive Airway Pressure: A Randomized Controlled Trial. Int. J. Prev. Med..

[34] Oncel M.Y., Arayici S., Uras N., Alyamac-Dizdar E., Sari F.N., Karahan S., Canpolat F.E., Oguz S.S., Dilmen U. (2016). Nasal continuous positive airway pressure versus nasal intermittent positive-pressure ventilation within the minimally invasive surfactant therapy approach in preterm infants: A randomised controlled trial. Arch. Dis. Child. Fetal Neonatal Ed..

[35] Sabzehei M.K., Basiri B., Shokouhi M., Naser M. (2018). A comparative study of treatment response of respiratory distress syndrome in preterm infants: Early nasal intermittent positive pressure ventilation versus early nasal continuous positive airway pressure. Int. J. Pediatr..

[36] Skariah T.A., Lewis L.E. (2019). Early nasal intermittent positive pressure ventilation (NIPPV) versus nasal continuous positive airway pressure (NCPAP) for respiratory distress syndrome in infants of 28–36 weeks gestational age: A randomized controlled trial. Iran. J. Neonatol..

[37] Shi Y., Tang S., Zhao J., Shen J. (2014). A prospective, randomized, controlled study of NIPPV versus nCPAP in preterm and term infants with respiratory distress syndrome. Pediatr. Pulmonol..

[38] Khorana M., Paradeevisut H., Sangtawesin V., Kanjanapatanakul W., Chotigeat U., Ayutthaya J.K. (2008). A randomized trial of non-synchronized Nasopharyngeal Intermittent Mandatory Ventilation (nsNIMV) vs. Nasal Continuous Positive Airway Pressure (NCPAP) in the prevention of extubation failure in pre-term <1500 grams. J. Med. Assoc. Thai..

[39] Kahramaner Z., Erdemir A., Turkoglu E., Cosar H., Sutcuoglu S., Ozer E.A. (2014). Unsynchronized nasal intermittent positive pressure versus nasal continuous positive airway pressure in preterm infants after extubation. J. Matern. Fetal Neonatal Med..

[40] Jasani B., Nanavati R., Kabra N., Rajdeo S., Bhandari V. (2016). Comparison of non-synchronized nasal intermittent positive pressure ventilation versus nasal continuous positive airway pressure as post-extubation respiratory support in preterm infants with respiratory distress syndrome: A randomized controlled trial. J. Matern. Fetal Neonatal Med..

[41] Komatsu D.F., Diniz E.M., Ferraro A.A., Ceccon M.E., Vaz F.A. (2016). Randomized controlled trial comparing nasal intermittent positive pressure ventilation and nasal continuous positive airway pressure in premature infants after tracheal extubation. Rev. Assoc. Med. Bras. (1992).

[42] Ribeiro S.N.S., Fontes M.J.F., Bhandari V., Resende C.B., Johnston C. (2017). Noninvasive Ventilation in Newborns </=1500 g after Tracheal Extubation: Randomized Clinical Trial. Am. J. Perinatol..

[43] Estay A.S., Mariani G.L., Alvarez C.A., Milet B., Agost D., Avila C.P., Roldan L., Abdala D.A., Keller R., Galletti M.F. (2020). Randomized Controlled Trial of Nonsynchronized Nasal Intermittent Positive Pressure Ventilation versus Nasal CPAP after Extubation of VLBW Infants. Neonatology.

[44] Kong L.K., Kong X.Y., Li L.H., Dong J.Y., Shang M.X., Chi J.H., Huang R.X., Zheng Y., Ma J.E., Chen X.C. (2012). Comparative study on application of Duo positive airway pressure and continuous positive airway pressure in preterm neonates with respiratory distress syndrome. Zhongguo Dang Dai Er Ke Za Zhi Chin. J. Contemp. Pediatr..

[45] Aguiar T., Macedo I., Voutsen O., Silva P., Nona J., Araujo C., Imaginário J., Mauricio A., Barroso R., Tomé T. (2015). Nasal bilevel versus continuous positive airway pressure in preterm infants: A randomized controlled trial. J. Clin. Trials.

[46] Sadeghnia A., Barekateyn B., Badiei Z., Hosseini S.M. (2016). Analysis and comparison of the effects of N-BiPAP and Bubble-CPAP in treatment of preterm newborns with the weight of below 1500 grams affiliated with respiratory distress syndrome: A randomised clinical trial. Adv. Biomed. Res..

[47] Pan R., Chen G.Y., Wang J., Zhou Z.X., Zhang P.Y., Chang L.W., Rong Z.H. (2021). Bi-level Nasal Positive Airway Pressure (BiPAP) versus Nasal Continuous Positive Airway Pressure (CPAP) for Preterm Infants with Birth Weight Less Than 1500 g and Respiratory Distress Syndrome Following INSURE Treatment: A Two-center Randomized Controlled Trial. Curr. Med. Sci..

[48] O’Brien K., Campbell C., Brown L., Wenger L., Shah V. (2012). Infant flow biphasic nasal continuous positive airway pressure (BP- NCPAP) vs. infant flow NCPAP for the facilitation of extubation in infants’ </=1250 grams: A randomized controlled trial. BMC Pediatr..

[49] Victor S., Roberts S.A., Mitchell S., Aziz H., Lavender T., Extubate Trial G. (2016). Biphasic Positive Airway Pressure or Continuous Positive Airway Pressure: A Randomized Trial. Pediatrics.

[50] Manjunatha C.M., Kalyanasundaram S., Ibhanesebhor S.E., Vigni D., Robertson C. (2019). Prospective randomized controlled trial comparing the use of biphasic positive airway pressure (BiPAP) with nasal continuous positive airway pressure (n-CPAP) following extubation of preterm babies. EC Paediatr..

[51] Kirpalani H., Millar D., Lemyre B., Yoder B.A., Chiu A., Roberts R.S., Group N.S. (2013). A trial comparing noninvasive ventilation strategies in preterm infants. N. Engl. J. Med..

[52] El-Farrash R.A., DiBlasi R.M., Abd E.L.A.E.A., El-Tahry A.M., Eladawy M.S., Tadros M.A., Koriesh M.A., Farid J.V., AbdElwahab R.S., Elsayed M.A. (2022). Postextubation Noninvasive Ventilation in Respiratory Distress Syndrome: A Randomized Controlled Trial. Am. J. Perinatol..

[53] John S.C., Mohammed A., Church J.T., John A.V., Perkins E.M., McLeod J.S., Carr B.D., Smith S., Barnett J.H., Gustafson P.A. (2021). Bubble bilevel ventilation facilitates gas exchange in anesthetized rabbits. Pediatr. Res..

[54] Badiee Z., Nekooie B., Mohammadizadeh M. (2014). Noninvasive positive pressure ventilation or conventional mechanical ventilation for neonatal continuous positive airway pressure failure. Int. J. Prev. Med..

